# Sexual health concerns in women with intellectual disabilities: a systematic review in qualitative studies

**DOI:** 10.1186/s12889-021-12027-6

**Published:** 2021-10-30

**Authors:** Behzad Karami Matin, Michelle Ballan, Fatemeh Darabi, Ali Kazemi Karyani, Moslem Soofi, Shahin Soltani

**Affiliations:** 1 Research Center for Environmental Determinants of Health, Health Institute, Kermanshah University of Medical Sciences, Kermanshah, Iran; 2 School of Social Welfare, Stony Brook University, New York, USA; 3 Department of Public Health, Asadabad School of Medical Sciences, Asadabad, Iran

**Keywords:** Sexual health, Sexuality, Sex education, Intellectual disability, Qualitative studies, Systematic review

## Abstract

**Background:**

Studies indicate that women with intellectual disabilities (ID) face various personal and socio-environmental barriers in their sexual lives. This study aimed to identify the concerns and sexual health needs experienced by women with ID.

**Method:**

A systematic review of relevant qualitative articles was conducted in PubMed, Web of Science Scopus and PsycINFO databases from June 2018 to August 2018. We designed our search strategy according to two main foci: (1) sexuality; and (2) women with ID. In the study, searches were limited to articles published from January 2000 to December 2017. In this review, studies on women ages 16 and over were included.

**Results:**

Within the four databases, the search found 274 unique articles. After three steps of screening (title, abstract and full text), 22 studies were included in the final review. The articles mentioned difficulties with lack of sexual experience, negative experiences with sexuality, negative attitudes towards sexuality by nondisabled individuals, limited cognitive capacities to understand sexual identity, difficulty with finding the right partner, lack of access to sexual health information, lack of school-based sexuality education, violence and sexual abuse, lack of support from families and caregivers about sexuality, fear of sexual acts and unwanted pregnancy, shyness in expressing sexual desires, and limited knowledge of sexual behaviors.

**Conclusion:**

Our findings indicate that women with ID need to be provided with school-based sexuality education tailored to the level of understanding needed to attain the requisite knowledge to form relationships, understand sexual and romantic relationships, and practice safe sex when they choose this option. Families along with education and healthcare systems should provide opportunities for women with ID to talk about their sexual needs and make their own choices.

## Background

Sexuality has a complex role in our lives and can influence one’s quality of life. Although sexuality can be a source of happiness and wellness, it also raises some ethical and cultural concerns in different societies [1]. According to the World Health Organization (WHO), sexual health is “the state of physical, emotional, mental and social well-being in relation to sexuality, and requires a positive and respectful approach to sexuality and sexual relationship.” The World Association for sexual Health, identifies different factors such as attitudes, sexual behaviors, societal determinants, biological factors and genetic disorders as influencing sexual health [2].

One group specifically impacted by lack of access to sexual health resources is women with intellectual disabilities (ID). ID is described as a disability of intellectual functioning and adaptive behavior that occurs during the period of time from conception to the beginning of adulthood. An individual With ID experience meaningful limitations in both intellectual ability and adaptive behavior that onset before the age of 18 [3]. Studies indicate that people with ID face various personal and socio-environmental barriers in their sexual lives. Some of these barriers include limited sexual knowledge, poor education, negative attitudes, lack of access to healthcare, lack of sexual experiences, and social isolation which can lead to increased sexual violence and abuse of women with ID [4–6]. Lack of awareness of sexual health can also result in sexually offensive behaviors among people with ID [7, 8]. Studies highlight that people with ID have problems expressing their sexual needs [9, 10]. Findings show that some people with ID do not have enough information about their sexual identity [11]. Lack of sexual health education contributes to increased prevalence of sexually transmitted infections among people with ID including an increased risk of HIV infection [11, 12].

Lack of knowledge about sexuality coupled with limited sexual experiences, language difficulties, communication problems, fear, embarrassment, low self-esteem and poor negotiating skills can increase exposure to unsafe situations for women and men with ID [13–15]. Esmail et al. note that the lack of access to information can perpetuate negative attitudes and misconceptions about women with ID [16]. These negative attitudes and beliefs can affect sexual functioning, intimate relationships, promotion of sexual health, safety, procreation, access to sexual health information and participation in sexuality education programs for women with ID [17–19].

Furthermore, healthcare systems can limit access to sexual health services for women with ID. Studies indicate that there is no consensus across providers on the approaches to sexuality education for this population [20]. For example, physicians are aware of sexually transmitted diseases among women with ID, however studies indicate they are less likely to have mammograms and pap smears compared to their nondisabled counterparts [21, 22]. Also administrative barriers such as funding shortages and lack of policy guidelines impact access to sexual health services for people with ID [20].

The sexuality of women with ID is not a new focus within the literature. However, access to sexual health services is an understudied and necessary foci in healthcare research for this population. Different studies have been conducted to identify the sexual health needs of both men and women with ID. In this study, we intend to provide a comprehensive overview of the challenges women with ID face regarding sexual health promotion. Thus, the main research question, according to PICOS, posed was: “What are the needs and concerns of women with ID (age>16 years old) regarding their sexual heath?” We examined the question from different perspectives (women with ID, families, and healthcare providers) to achieve a more complete and deeper understanding of the role of sexual health promotion among women with ID, with specific attention to the unique challenges to access sexual health services in low and high income countries. Through this review, we hope the findings are helpful to researchers, policy makers, providers, self-advocates and other stakeholders who address the needs and concerns of women with ID and the barriers they face.

## Method

### Selection of studies

To investigate and identify the sexual health needs of women with ID, we decided to include only qualitative study designs in this review. There were three reasons to focus on this type of study. First, qualitative studies can provide a deep understanding of participant’s attitudes, beliefs, interactions, experiences and behaviors [23]. Second, some concerns and needs in sexual health may be unique among the participants that are not reflected completely in quantitative studies. Third, in qualitative studies, researchers are able to evaluate human behaviors more extensively than quantitative studies [23]. Thus, the observational studies (case-control, prospective, cross-sectional studies), experimental (quasi-experimental and randomized controlled trials), and review articles (narrative, scoping and systematic reviews) were excluded. In this study, young women with intellectual disabilities (older than 16) were included. Sixteen was selected to represent the age of consent for sexual relations in many countries, however the age to consent in some countries or states may be lower or higher.

**Fig. 1:**
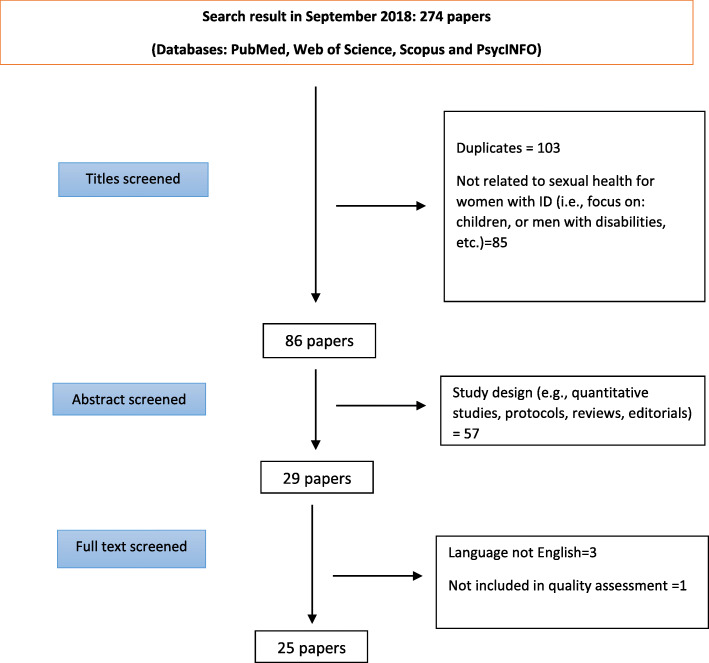
The process of systematic review of the literature

In addition, we excluded non-English language studies and grey literature (such as books, dissertations, conference abstracts) from the research. For studies without full text access, we sent an email to the authors and asked them to send the article. Other criteria to include and exclude studies has been provided below. Furthermore, the process of systematic review of the literature is shown in Fig. 1. In some studies men and women with ID had participated in the study. We included these studies as well and extracted only the women’s quotations.

According to PICOS, the related criteria to include studies were:

1. **Population**: Women with ID 16 years of age or older
2. **Intervention**: N/A
3. **Comparison**: N/A
4. **Outcome**: concerns and needs related to sexual health among women with ID
5. **Study design**: Qualitative studies
6. Published in English between January 1, 2000 and December 31, 2017
7. Full-text articles

The related criteria to exclude studies included:

1. Published before January 1, 2000 and after December 31, 2017
2. Abstracts, editorials, letter to editor, and comments
3. Protocols and method papers
4. Quantitative studies
5. Studies on individuals with ID under the age of 16
6. Grey literature (e.g. books, conference abstracts, theses, research reports, policy documents)
7. Non-English language studies
8. Studies that were not related to sexual health

### Search strategy

This structured review was conducted using the guidelines of Preferred Reporting Items for Systematic Reviews and Meta-Analysis (PRISMA). The literature search included four bibliographic databases; “Web of Science”, “PubMed”, “Scopus” and “PsycINFO” involving all of the related studies from January 2000 to December 2017. Searches were conducted from June to August 2018. The studies were searched based on two main foci: (1) sexual health; and (2) women with ID. Our keywords to search articles were a combination of the words sex, sexual activity, sexual abuse, disability, intellectual disability, developmental disability and qualitative. Moreover, hand-searching reference lists of the included papers was applied to further identify articles which met our inclusion criteria. The results of our search procedure in the four selected databases were 274 references which were exported to EndNote software version X7. The search strategies to find the relevant studies is shown in Table 1.

**Table 1:** The search strategies^a^ to find the relevant studies

Search Strategies	number
**Web of science**	
**TOPIC:** (disabilitya) *AND* **TITLE:** (sex^a^) *AND* **TOPIC:** (qualitative^a^)	105
**TITLE:** (intellectual disability^a^) *AND* **TITLE:** (sex^a^) *AND* **TOPIC:** (qualitative^a^)	20
**PubMed**	
((intellectual disability[MeSH Terms]) AND sexual activity[MeSH Terms]) AND qualitative[Title/Abstract]	14
((intellectual disability[MeSH Terms]) AND sexual behavior[MeSH Terms]) AND qualitative[Title/Abstract]	14
((intellectual disability[MeSH Terms]) AND sexual abuse[MeSH Terms]) AND qualitative[Title/Abstract]	4
**Scopus**	
(TITLE (disability) AND TITLE (sex) AND TITLE-ABS-KEY (qualitative))	18
TITLE (intellectual AND disability) AND TITLE (sex) AND TITLE-ABS-KEY (qualitative)	11
mainsubject(disability^a^) AND mainsubject(sex^a^) AND ab(qualitative^a^)	62
ti(disability^a^) AND ti(sex^a^) AND ab(qualitative)	26
**Total**	274

^a^Regarding that each database provide different strategies to search articles, we used different key words and adapted our search strategy for each database

### Data extraction

We set up a checklist in which the needed information such as authors, year, country, methodology, samples, study perspective, themes and main findings were extracted from the included studies. Searching and identifying the studies was executed by the corresponding author. The third and fourth authors of the present study (FD and AKK), summarized the findings and categorized the required information according to the prepared checklist. To ensure the validity of the checklist information, the corresponding author (SS) checked the accuracy of the extracted data. We identified all disagreements and resolved them by consensus after discussion. Also, we used MAXQDA version 11 to code and categorize qualitative data.

### Quality assessment

Quality assessment of qualitative studies is a frequently debated topic in the literature. Contrary to qualitative studies, determining reliable criteria to assess the quality of qualitative studies may be a complicated problem for researchers. Given the aim of the study, we assessed the quality of the included studies using the Consolidated Criteria for Reporting Qualitative Research (COREQ) [24]. The quality assessment included the topics of study design, research framework, sampling method, gathering appropriate data according to aim of the study, data analysis approach, appropriate categorization of findings, reporting the findings and related data (photographs or quotes), etc. Two independent researchers who had no additional contribution to this study assessed the quality of included studies using a 5 point Likert scale. In order to apply the COREQ in the review process, we held two training sessions for the researchers. All items in the checklist were assessed and scored (from 1 to 5) by both researchers separately and the mean score was determined as the final score of the quality assessment. Therefore, in the final step of the review, we included studies that earned 3 points or higher.

## Results

According to the stipulated research strategy, the corresponding author identified 274 records in the four databases, imported them into Endnote software and screened them according to the inclusion and exclusion criteria. The first step of screening was done according to the title of the studies and 197 articles were removed due to duplicates and the date of publication (before January 2000 or after December 2017). At the next step, the abstracts of the papers were studied and we excluded 47 additional papers due the method or study design (quantitative studies, review studies, letter to the editor, editorial, and protocols). At the final step of the screening process, we studied the full text of the remaining articles to extract their findings. In this step, we included 25 qualitative studies in the final step of the review process. Of these, three articles were unavailable in full text. The authors were contacted by email and they provided the articles. All 25 articles were categorized according to geographical area; 12 studies were conducted in Europe, 3 in Asia, 4 in the North America, 4 in Australia and 2 in Africa. Table 2 shows the characteristics of the reviewed studies.

**Table 2:** Characteristics of the included studies

Authors & year	Country	Nmber of participants	Sampling method	Age	Perspective	Study design(location of data collection)	Study aim	Main findings	
								• Concerns	• Needs
Darragh et al. (2017) [25]	Australia	Number(N): 30(Males(M): 22, Females(F): 8)	Not mentioned (NM)	20–66 years	People with ID	semi-structured interviews(participant’s home or place of employment)	To explore if people with intellectual disability use social media to form relationships that express their sexuality	• Creating new friendships • Maintaining existing friendships • Expressing sexuality • Being worried about assessing, managing, and mitigating the risks relating to meeting a partner for the first time	• Receiving formal education • Being supported by family
Bjornsdottir et al. (2017) [26]	Iceland	N: 29(M:10, F:19)	Maximum variation sampling strategy	26–66 years	People with ID	semi-structured individual interviews and focus groups(participants’ homes)	To addresses the manifestation of masculinity, femininity, and autonomy in the lives of people with ID	• Being surveilled and controlled by staff and family members • Limited opportunities in the institutions to develop one’s autonomy • Sterilizing women with ID • Worrying about become pregnant • Sexual abuse or harassment • Not receiving any support or counseling following the sexual abuse • Being viewed as an emotionless person • Being controlled by staff and family (childlike) • Lack of sexuality education • Being prohibited from participating in sexuality education • Limited knowledge regarding sexual health	• Being independent in decision making • Normalizing gender/sexuality in the lives of people with ID in schools and health systems • Developing autonomy
Frawley and Wilson (2016) [27]	Australia	N:25 (M:14 F:11)	NM	17–20 years	People with ID	Focus group and individual interviews (NM)	To explore young adults with ID talking about sexuality education and information	• Lack of knowledge about how to have sexual intercourse • Lack of access to information	• To know more about their sexuality
Schaafsma et al. (2016) [28]	Netherlands	N: 20 (F:20)	NM	15–52 years	People with ID	Semi-structured interviews (participant’s home or institution)	To assess the perspectives of people with ID on sexuality-related topics.	• Lack of knowledge of condom use • Incomplete knowledge of contraception • Lack of awareness of how condoms protect • Lack of knowledge about homosexuality • Concerns regarding negative experiences and sexual abuse • Carers’ interference in intimate relationships	• Sex educationtopics and knowledge • To have a partner • Expressing sexual experiences • To have a child and have children • Social media’s role • Providing special supports by carers
Turner et al. (2016) [29]	United States	N: 5 (F:5)	Purposeful sampling	21–70 years	People with ID	interview and Observation(participant’s home or author’s therapy office)	To explore how adults with mild (ID) experience their social-sexual lives.	• Lack of training programs • Lack of contraceptive programs • Worrying about isolation • Being Concerned about intimacy • Anxiety about vulnerability and risk-taking	• Intimacy (i.e. emotional pleasure) • Sexual experience • Improving sexual attitudes • Social justice • Friendship and marriage • Recognizing emotional and physical pleasure • Understanding sexual self-identity
CHOU et al. (2015) [30]	Taiwan	N: 11 (M:6 F:5)	Purposive sampling	18–63 years	People with ID	Focus groups(home or residential setting)	To explore attitudes toward sexuality among men and women with ID in Taiwan	• Feeling embarrassed to talk about sexuality-related issues • Lack of parents’ desire to talk about sexual health issues • Bing Shy in expressing feelings • Negative life experiences concerning sexuality • Being raped by friends • Being inappropriately watched during showers • Being forcibly kissed in the workplace • Being inappropriately touched on the bus • Difficulty finding a partner • Receiving inadequate parental support • Having to hide romantic relationships	• Developing intimate relationships • Receiving support to get married
Lofgren-Martenson (2004)^15^	Sweden	N: 36 (13 youths and young adults, 13 staff members and 11 parents)	NM	16–21 years	Women with ID Health care providers Families	Interview and observation (special schools)	To identify, describe and understand the opportunities and hindrances for young people with ID regarding sexual expression such as homosexuality and bisexuality.	• Problems with communication and language ability • Receiving little education about sexuality • Difficulty with understanding education • Difficulty with expressing views • Difficulty with specifying the needs (due to lack of knowledge) • Worrying about Negative effects of childbirth films on sexual desire • Loneliness, alienation and bullying • Being shy • The risks of seeing pornography • Problems with remembering previous sexual educations • Being embarrassed to talk about sexuality • Difficulty understanding, concentrating and focusing	• Sexual education in schools, especially in relation to homosexuality and heterosexuality • Sex education about feelings and relationships • Sex education regarding the body’s functions particularly in special education • Education regarding safer sex and the use of contraception • Education about relationships, love and friendship • Sex education at younger ages • Opportunities to speak to a trusted teacher about sexual health • Broadcasting TV shows about sex and relationships • Learning in different ways, such as reading books, watching films, role playing and discussion
Rushbrooke et al. (2014) [31]	UK	N: 9 (M: 5 F:4)	Purposeful sampling	21–58 years	People with ID	Semi- structured Interviews (home or supported accommodation)	To carry out an interpretative phenomenological analysis exploring the experience of intimate relationships for nine adults with ID	• Fear and embarrassment relating to sexual expression • Sexual identity or orientation • Family disagreement about being in relationships • Caregivers’ reactions about being vulnerable’ and susceptible to risk • Struggles with searching and finding a partner • Feelings of frustration and upset about finding a partner • Being controlled by family, caregivers and society	• Having a partner • Expressing sexuality • Being independent • Having opportunities to meet people • Starting a relationship • Having a relationship and ending a relationship
Azzopardi-Lane and Callus (2014) [32]	Malt	N:7 (M: 3 F: 4)	NM	20–59 years	People with ID	Focus groups(the Consultative Committee of Persons with Intellectual Disability (KCC))	To explore the perceptions of people with ID about sexuality and how these are influenced by social and cultural norms	• Not having a relationship because of disability • Feeling constrained because of lack of privacy, limited finances, as well as reliance on others for support, including transport • Being controlled by parents and carers • Embarrassment about the subject of sexuality • Lack of exposure to sexually related conversations and images • Negative reactions from public related to sexual expression • Lack of consent from parents • Lack of trust among parents • Worrying about unwanted pregnancy among parents	• Talking about their sexuality and the type of relationships they would like to have • Having more opportunities to have sexual relationships • Going out with an intimate partner and getting married • Having opportunity to socialize with their counterparts • Support from family members • Being more independent and autonomous • Regarding the rights of people with ID
Thompson et al. (2014) [20]	Australia	N: 31 (disability service manager:8, Clinicians: 23)	Purposive sampling	Not Applicable (NA)	Health service providers	Semi-structured interviews(clinic)	To explore barriers to sexual health provision for people with ID	• Worrying about being abused • Lack of information and education about sexual health • Limited sexual health resources • Lack of training for staff and health services providers • Lack of positive attitude toward sexuality of people with ID	• Providing financial resources for sexual health services • Formulating policies on sexual health provision • Respect for privacy • Understanding effectiveness of sexual knowledge assessment tools • Increasing awareness among clinicians to address the sexual needs of people with ID • Identifying barriers to provide sexual health services
Rojas et al. (2016) [33]	Spain	N: 16(M: 10, F: 6)	NM	18–39 years	People with ID	Semi-structured Interviews (NM)	To examine sexuality and intimacy in the lives of people with IDs	• Receiving Negative reactions from families • Receiving limited training • Lack of knowledge and skill • Misconceptions about females with ID • Being controlled by staff and families • Lack of privacy • Feeling worried about breaking family rules	• finding a partner and living together as a couple • Being supported by family • Receiving information about sexuality • Being independent
Bernert and Ogletree (2013) [1]	United States	N:14 (F: 14)	NM	18–89 years	People with ID	In-depth interviews and observation (the local agency)	To examine sexuality in the lives of women with IDs	• Having limited knowledge of sexual behaviors • Fear of intercourse and intimacy • Having limited and exclusively heterosexual experiences • Practicing abstinence • Feeling fear of the first sexual act • Feeling scared of negative consequences of sexual intercourse • Having physiological concerns about the sexual act • Perceived or actual lack of pleasure • The absence of language to talk about intercourse or discomfort • Worrying about unintended pregnancy • Worrying about sexually transmitted diseases	• Having protected sex • Having monogamous sex for the purpose of procreation or parenting • Caring for loving a sexual partner
Stoffelen et al. (2013) [34]	Netherlands	N: 21 (M: 19 F:2)	NM	20–62 years	People with ID	Semi-structured interviews (the Dutch Gay, Lesbian, Bisexual, and Transgender Organisatio)	Identifying sexual experiences of homosexual people in the Netherlands with mild ID	• Negative sexual experiences • Experiencing sexual abuse • Difficulty to be open about their homosexual identity • Being unable to talk about sexual orientation • Feeling embarrassed to provide sexual guidance among caregivers • Limited support from caretakers to find a partner • Lack of privacy	• Receiving specific training programs on sexuality • Safe work and living environments • Identifying meeting places • Supporting people with ID to identify and prevent socially (un)acceptable situations • Being supported to seek a new partner
Aderemi (2013) [35]	Nigeria	N: 12 (M:3 F:9)	Purposive sampling	20–62 years	Health Service providers	Individual interviews (special schools)	This paper reports on teachers’ opinions on sexuality of Nigerian learners with ID and awareness of their risk of HIV infection.	• Lack of skills to provide sexuality and HIV education by providers • Lack of sexuality related curriculum for special schools • Feeling fear of sexually transmitted infections • Lack of sexuality and HIV information in accessible formats • Lack of capacity for intimate relationships	• Receiving sexuality and HIV prevention education • Preparing teachers to teach sexuality and HIV education
Lafferty et al. (2012) [36]	Northern Ireland	N:96 (Family Carers: 48 Health Service providers: 48)	Snowballing recruitment	NA	Family and Service providers	Group and individual interviews” (voluntary organizations)	To identify the barriers to relationships and sexuality education for persons with intellectual disabilities	• Lack of training • Mental health problems • Getting sexually transmitted infections • Inadequacy of education in schools • Cultural prohibitions • Scarcity of educational resources • Showing abnormal behaviors in public • Facing religious beliefs	• Protection and safety • Providing sexuality education in schools • Receiving information about pregnancy, sexually transmitted infections, and contraception
Eastgate et al. (2012) [37]	Australia	N:28 (F:28)	NM	> 18 years	Family members and support workers	Semi- structured interviews and focus groups (community organizations and clinics)	To identify the problems of women with ID in sexuality, relationships and abuse prevention	• Simplistic and unrealistic ideas of relationships • Disempowerment • Sexual exploitation via the internet and mobile telephones • Exploitation and sexual abuse such as coercion and manipulation • Poor understanding of sexuality • Lack of knowledge about sexuality issues • Negative attitudes toward educating people with ID	• Supporting safe, constructive sexual relationships
Nareadi Pasha and Nyokangi (2012) [38]	South Africa	N:16 (F: 16)	Purposive sampling	16–24 years	People with ID	In-depth face-to-face interviews (schools)	To identify school-based sexual violence experiences of women with mild ID	• Sexual violence such as touching, threats and intimidation • Coercive sex or rape • Feeling scared to report sexual abuse to the family or the police • Viewing rape as a strategy to punish an intimate partner • Receiving sexual pictures and pornographic magazines • Misconceptions about sexual performance of females with ID	• Providing appropriate sex education programs • Increasing self-protection skills • Access to different forms of information • Care and support for victimized females with ID • Formulating explicit policies and programs on sexual abuse
Fitzgerald and Withers (2011) [39]	UK	N: 10 (F:10)	NM	19–64 years	People with ID	Semi-structured interview (clinics)	How women with ID conceptualize their sexuality or develop a sexual identity	• Talking about sex and their sexuality • Ignoring sexual needs • Being worried about homosexual tendency • Prohibition of sexual expression • worrying about sexual intercourse • Feeling fear of financial exploitation	• Talking about sex and sexuality • Experiencing sexual desires • Increasing awareness of contraception among general practitioners, staff and parents • Having safety in intimate relationships
Chou and LU (2011) [40]	Taiwan	N:11 (family member: 7, Female with ID: 4)	NM	20–58 years	Family and people with ID	Semi-structured interviews (family homes)	To explore decision-making regarding sterilization for women with ID living with their families	• Decision making by husband or parents-in-law • Being unable to care for the children • Risk of pregnancy from rape • Being concerned about genetic disorders in children • Difficulty of menstrual care	• Having autonomy to engage in decision-making • Attention to human rights • Access to sexual health information • Providing educational programs • Receiving more supports from families and staff
Eastgate et al. (2011) [41]	Australia	N:9 (F:9)	Snowballing sampling	> 18 years	People with ID	Semi structured interviews (community organizations)	To explore how women withID understand sex, relationships and sexual abuse, the effects of sexual abuse on their lives, and how they successfully protect themselves from abuse	• Unwanted or abusive sexual acts • Feeling Fear of sex and avoidance of relationships • Difficulty refusing unwanted relationships • Returning to abusive situations • Sexual abuse by fellow students • Limited understanding of sex • Inadequate self-protection skills • Difficulty reporting abuse and obtaining appropriate support • Lack of literacy and skills to seek information independently • Absence of sexuality education at school • Forgetting what has been learned at school	• Being aware of the possibility of sexual abuse by general practitioners • Providing ongoing training regarding sexual health for women with ID • To be asked directly about sexual relationships • To facilitate access to information or support • Managing complex mental health and behavioral challenges • Providing innovative programs • To be Protected from abuse
Swango-Wilson (2011) [42]	United States	N: 3 (F:3)	NM	Not Specified	People with ID	Individual interviews (local agencies)	To identify what individuals with ID/DD expect from a sex education program	• Lack of knowledge about sex education • Fear of getting pregnant • Lack of social skills	• Providing sex education • Methods of instruction for sex education learning • Trust, reliability, and the ability to talk about problems • Finding committed sexual partner • Increasing parents’ awareness of marriage of children • Increasing awareness of safe intimacy • Increasing awareness of contraceptive methods • To have mixed gender sex education classes to understand different ideas • Providing sex education programs
Healy et al. (2009) [43]	Ireland	N: 32 (F:32)	NM	18–30 years and 31<	People with ID	Focus groups (community based services)	This study sought to gather information from people with ID about their nowledge, experiences and attitudes towards sexuality	• Social isolation • Lack of knowledge about masturbation, sexual consent, safe sexual activities, and The use of condoms in preventing sexually transmitted diseases (STDs) • Lack of awareness of HIV transmission • Poor knowledge of both STDs and male/female sexual anatomy • Lack of privacy in the institutions • Feeling afraid to talk about sex and related issues among carers	• Having personal relationships • Companionship and security in relationships • Respect and privacy • Sexual skills training for carers • Learning about sex through formal sex education • Learning about contraception and its role in preventing pregnancy • Increasing knowledge of STDs • Having right to Marriage and having a child
Kwai-sang Yau et al. (2009) [44]	Hong Kong	N:12 (M: 3 F: 9)	NM	22–44 years	People with ID	Individual Interviews (NM)	To explore sexuality and sexual concerns of people with ID in a Chinese cultural context	• Lack of awareness among school and family members about sex education • inappropriate sexual behaviors in the community • Lack of awareness about gender roles in the community • Sexual attitudes (sexual harassment, sexual exploitation, rape, unwanted pregnancy, and health hazards such as STDs)(	• Dating and intimate relationships • Enhancing sex knowledge • Positive family attitudes regarding sexual desires of women with ID • Striving to live normal lives
Kelly et al. (2009) [18]	Ireland	N: 15 (M:8 F:7)	NM	23–41 years	People with ID	Focus group (disabilities service)	To examine experiences and needs with respect to sexuality and romantic relationships among women with ID	• Lack of sex educationfor people with ID Confidence in relation to the opposite sex • Lack of awareness of rights in relation to their sexuality • Random and opportunistic ways to acquire information about sexual issues	• To acquire information about sexuality and intimacy • Having opportunity to express ideas and have friends • Having romantic relationships
McCarthy (2009) [17]	UK	N: 23 )F: 23(	Purposive sampling	20–51 years	People with ID	Interview (community-based settings)	To examine experiences and needs with respect to sexuality and romantic relationships among women with ID	• Lack of knowledge about menopause • Pregnancy during sexual activity • Lack of awareness of unwanted pregnancy	• Awareness about women’s contraceptive methods • contraception • Knowledge regarding family planning and contraception

In the included studies, Participants were recruited from different settings such as censuses and surveys [40], community-based services (e.g. community learning disability services [31, 33, 43], clinics, organizations (e.g. voluntary organizations, organizations specialized in care for people with intellectual disabilities, the Dutch Gay, Lesbian, Bisexual, and Transgender Organization, voluntary organizations, special schools [34–36, 45], agency professionals [29], self-advocacy groups (e.g. Consultative Committee of Persons with Intellectual Disability) [32]. Some participants lived with their parents, some used residential services (i.e., group home), and some lived independently with support staff visiting for a number of hours a week [14, 15, 34, 44, 46].

Marital status for women with ID was various so that some of them were married, some were single, some reported being divorced, some were widowed, and some of them had biological children [32, 33, 45]. Some women with ID were employed during the study [1, 26, 41, 47]. Data collection was done in different setting such as participants’ homes, offices, clinics, or residential settings [14, 20, 26, 28, 45]. Overall, different groups such as women with ID, parents, support workers [37], caregivers, teachers [35], social workers, educationalists, professionals (e.g. psychologist, occupational therapist) [36], and social care managers, nurses [36] participated in the included studies. Five studies reported the severity of ID so that they included women with mild or moderate ID in their studies [17, 29, 30, 34, 38]. Also, in most included studies, women with ID were eligible to participate in the study if they had a diagnosed intellectual disability. None of the included studies mentioned the cause of ID.

Women with ID mentioned different concerns in the included studies. We labeled each concern as a separate descriptive code. Codes were then used to summarize segments of data for each article, and we compared codes and sorted them into categories. The categorization of the concerns stated by participants is shown in Table 3.

**Table 3:** The categorization of the concerns mentioned by participants in the included studies

Category	Examples	Number of codes
Being controlled	Being monitored by staff in instructions, lack of privacy	**6**
Education	Inadequacy of school-based sexuality education, being prohibited from participating in sexuality education, lack of training programs for staff and families	**9**
Knowledge and skills	Limited knowledge of STDs, unwanted pregnancies, rights, genders roles, sexual identity, self-protection, female and male anatomy, and contraceptive options	**24**
Sexual abuse	Sexual abuse in different ways (i.e., internet, manipulation, verbal) and in different settings such as public places, difficulty reporting abuse, difficulty refusing unwanted sexual intercourse, sexual harassment, fear of financial exploitation	**16**
Information	Lack of literacy and needed skills to seek information independently, limited sexual health resources	**5**
Lack of support to find a partner	Limited support from caretakers, family disagreement, receiving less parental support, reliance on others for supports, lack of trust among parents, lack of consent from parents, parents’ concern about getting pregnant, lack of support to engage in a relationship and access to a meeting place	**9**
Shyness	Loving person in secret, being embarrassed to talk about sexual issues, shyness in expressing feelings	**10**
Anxiety and fear	Fear of sex, fear of first sexual act, concern about unwanted pregnancy, concern about isolation and sterilization, psychological concerns, concern of negative sexual experiences, fear of STDs,	**26**
Communication	Difficulty with expressing views and specifying needs, not being able to talk about sexuality	**6**
Sociocultural barriers	Social isolation, prohibition of sexual expression, negative reactions from staff, families and public, being viewed as an emotionless, being sexually inactive, religious beliefs,	**9**
Limited experiences	Lack of exposure to sexuality, Lack of pleasure	**3**
Poor understanding	difficulty with understanding sexual orientation, societal norms, emotional feelings, sexual needs	**6**
Intellectual capacities	Forgetting what has been learned, not being in a relationship due to disability, inappropriate sexual behaviors, problem with remembering issues	**6**

### Knowledge and skill

Across the studies, participants identified lack of sexual knowledge as a main barrier for women with ID to experience a healthy sexual relationship. Limited knowledge of contraceptive methods, sexual behaviours, sexual abuse, and the process of sexual intercourse and pregnancy were reported. In most studies, participants had a simplistic understanding of the process of sexual intercourse yet, in a limited few, women with ID possessed a sophisticated understanding of sexual intercourse. For example in Eastgate et al.’s study, some participants with mild ID had correct information about menses, time of ovulation and sperm motility. In others, participants lacked knowledge of the basics about males’ bodies and some women with ID did not know how to develop a sexual relationship with men [27]. For example, some participants had problems defining the progression from kissing or touching to a penetrative relationship.

### Education

One of the major reasons for limited knowledge of sexual health can be attributed to the lack of access to comprehensive sexuality education. The review of findings indicated that the absence of sexuality education was a serious concern and detriment to the sexual health for women with ID. Some participants in the studies explained that they have not received any school-based sexuality education. For example, they were excluded from the classroom during sexuality education lessons. Women with ID in some schools were forbidden to talk about sexuality as it was considered an illegal act for them [41]. Some remarked that sexuality education was very general and thus they were not able to obtain complete information about their sexuality and bodies [26]. Also, participants noted that as adults, they need sexuality education to form romantic relationships and to become parents. Regarding the needs mentioned by women with ID, this education can be provided by knowledgeable parents, teachers and health professionals and consistently reinforced.

### Sociocultural barriers

Some participants had to hide their sexual experiences and their consensual sexual contact in institutions or group homes because of prohibitive rules, negative attitudes towards their sexuality and lack of privacy. Stoffelen et al. found that women with ID desiring same-sex relationships experienced difficulty talking about their sexual orientation freely [34]. In Berenert et al.’s study, participants had a negative perception of sexual intercourse [1]. Engaging in some sexual acts was physically painful for them and the frequency of sexual intercourse was not a pleasant experience for some participants. For example one of the women described sex as “Sickening. That’s how I think of it anymore. It’s disgustingly gross, all [he] wanted was sex, but I didn’t like havin’ it all sex, all the time” (p. 245) [1]. Some participants were not curious about sex and felt regret having sex at younger ages because they were not ready to have a sexual relationship [1, 30, 48]. Some participants described sex as ‘ugly, dirty, bad, disgusting, and displeasurable’(p. 245), and worried about their first sexual intercourse in the future, stating that it would be scary [1]. In addition, they were worried about the sexual health problems of their partner. Women with ID were scared of the negative consequences of sex like unintended pregnancy or sexually transmitted infections. Unwanted pregnancies in sexual intercourse between people with ID was one of the major concerns of families [37, 49, 50].

### Being controlled

Many of the women with ID noted that they had little control over their lives. Lack of autonomy caused limited opportunities for women with ID to form friendships and romantic relationships. Women with ID living in institutions had to behave according to the institution’s rules. In Kelly et al.’s study women with ID noted that their relationships with partners are controlled by staff in the institutions and thought that they were not trusted to have a healthy romantic relationship [18]. Also, in Bjornsdottir et al.’s study, which included both male and female participants, women seemed to be controlled by families and staff for sexual activities, fertility and sterilizations at a greater degree than males [26]. For example one of participants in the included studies stated that “I wasn’t allowed to move to the group home unless I had the sterilization” (p. 304). Not only were the participants’ decisions and choices about an intimate relationship restricted by families or caregivers but also the lack of services and stigmas perpetuated by society delimited their options. They noted that people think that they cannot have a sexual or friendly relationship because of ID [31]. Also, they mentioned that being controlled by families has affected the quality and stability of their relationships with their boyfriends. This problem would lead to dissatisfaction among women with ID so that they were not able to express their sexuality openly. Also, Rushbrooke and colleagues identified some internal and external factors contributing to a lack of control by women with ID [31]. Internal factors included being passive in relationships, perceived ability to cope, and insufficient opportunities to create social contacts and spend time with partners. External factors were categorized as the influence of others and the impact of policy. The participants stated that being controlled along with negative experiences caused a lack of confidence and anxiety.

### Lack of support to find an intimate partner

One of the important needs of women with ID to have a safe sexual relationship was finding an intimate partner. In Stoffelen et al.’s study, although the participants reported they desired a partner, they did not know how to find one [34]. They need support to seek a partner but their caregivers did not know how to provide proper support. Women with ID need opportunities and venues to socialize to provide opportunities to meet an appropriate partner. In Chou et al.’s study, participants mentioned that they were attracted to and had feelings for their teachers or male relatives in secret. For example one of the women with ID stated that “I have liked my teacher at the gas station (workplace) for two years. But I am shy to tell him” (p.672) [30]. Furthermore, women with ID experienced problems with ending an intimate relationship when they did have a partner. Some women with ID reported that they have boyfriends but they need supports to find a place to make love [34]. Additionally, discrimination in providing supports was one of the concerns mentioned by women with ID. They noted that men with ID faced less barrier to get married and their families agree to the marriage of their children [31].

### Sexual abuse

In the included studies, many of the participants had stated negative experiences concerning sexuality in their lives. Sexual abuse by friends and colleagues frequently was reported by women with ID. Being forcibly kissed, viewed or touched inappropriately, and raped were experiences reported by women with ID [30]. Sexual abuse usually happened in public places like institutions, schools, public transportation, and workplaces [30, 31, 41]. When reporting sexual abuse to police or other authorities, women with ID encountered negative reactions from their families or caregivers. Some of the interviewees had experienced sexual abuse beginning as involuntary sterilization during their adolescence.

Vulnerability was one of the considerable concerns mentioned by women with ID or their caregivers. In some studies, the limited knowledge of and the lack of skills needed for healthy sexual contacts would make them especially vulnerable to sexual abuse. Eastgate found that disempowerment can increase the probability of sexual exploitation. For instance the mother of a woman with IDs stated that “I think she would just want to make that person happy and do whatever they want her to do … she does things to please people”(p.136) [37]. In the included studies, a variety of unwanted actions such as manipulation and coercion were mentioned as sexual abuse by women with ID. Some studies reported that emotional relationships that occur in secret may increase the risk of sexual abuse for women with ID. But the findings of Schaafsma et al. show that public places like schools and institutions can leave women with ID more vulnerable to different types of physical and verbal abuse. In this particular study, women with ID who had experienced sexual abuse stated that they were targeted because they had been sterilized and thus could not become pregnant as a result of the abuse [28]. As a result, the experience of sexual abuse would lead to fear of new emotional relationships among participants in the future.

### Communication

Due to a speech and cognitive impairments, women with ID faced problems to talk about sexuality issues. They were not able to explore and specify their sexual needs. Some participants had problems recalling previous learning about sexual topics [41]. Cognitive challenges affect social interactions among women with ID. Some women with ID reported that they were not able to express their feelings and sexual needs [45]. In the included studies, some participants noted that women with lower intellectual functioning had less of a chance to create and maintain an intimate relationship [35, 51].

### Shyness

The feeling of embarrassment was one of the considerable problems of women with ID to find a partner and create an intimate relationship. The attribute would affect sexual expression among women with ID. In some studies, participants were shy to discuss about topics like masturbation, sexual intercourse and sterilization [30]. Some interviewees with ID preferred to attend classes with a familiar teacher or only with a woman to receive sex education. In Löfgren-Martenson’s study, one of the participant noted that she never talk to anyone about sexuality and this was a painful problem for her [45]. In addition, some studies indicated that some parents and carers are shy to provide sexual guidance for women with ID [31, 32, 34].

### Information

Lack of information in relation to sexuality was one the concerns mentioned by women with ID in the included studies. Because of limited intellectual capacities they faced barriers to seek the needed information. Also, lack of literacy and the communication skills such as listening, eye contact and empathy would exacerbate their access to sexual health information [41]. They received their information randomly through their friends or relatives [18]. Thompson et al. found that limited sexual health resources was one of the major barriers to access the needed information among women with ID [20].

## Discussion

The aim of the systematic review study was to identify sexual health needs experienced by women with ID worldwide. The findings of the study indicated that women with ID have sexual desires and need specific supports to make a healthy sexual relationship. Lack of education was a major problem stated by participants in the included studies. They need to be engaged in sexuality education programs with trusted caregivers to improve their knowledge about sexual health. Sexuality education programs need to be adapted to the needs of women with ID. Studies show that sex education interventions can have a positive impact on knowledge, self-protection, empowerment, and decision making for women with ID [47, 52]. Sexuality education should cover different dimensions including contraceptive methods, self-protection skills, sexual consent, and STI. It is important to note that caregivers and family members can play a considerable role in sexual health education. Women with ID often have a poor understanding of sexual knowledge that can lead to inappropriate behaviors and increase the likelihood of sexual abuse [37, 43, 53]. Learning how to meet partners, engage in healthy relationships and appropriately express romantic feelings could help to minimize these negative outcomes [18, 30, 31].

In the studies reviewed, some women with ID reported being forbidden to participate in sexual education in schools [26]. Cultural prohibitions sometimes limit access to sexual care and increase unmet sexual health needs for women with ID [36]. The findings reveal that sexuality issues are a taboo subject increasing the challenges to seeking information.

Finding an intimate partner is a notable issue mentioned by women with ID in the included studies. Although the studies showed that women with ID would like to engage in romantic relationships, participants reported difficulty expressing their feelings. Lack of confidence, shyness, families’ dissatisfaction, and lack of a meeting place were leading factors that affect their relationships. Studies reveal that parents have more conservative attitudes toward sexual activities in women with ID [54, 55]. In the Swango-Wilson’s study, some participants mentioned that commitment is an important issue in developing a lasting relationship with men but some of them were not ready to make a commitment to start a family [53]. According to the above-mentioned studies, women with ID should receive more support from their families and caregivers to develop or end an intimate relationship. Participating in educational and supportive programs can help promote constructive options to develop healthy relationships. Families need to provide more opportunities for women with ID to express their feelings. Respecting the feelings and desires of women with ID should be considered by families and policymakers. Also, cultural barriers like discriminations and misconceptions need to receive more attention by researchers especially in countries with more religious restrictions.

The present review indicated that women with ID have little control over their lives. They were not able to make independent decisions about their sexual lives. In residential facilities, women’s fertility is controlled by families and governmental policies. This study indicates that many women with ID do not have access to necessary information to develop autonomy in their private lives [46]. Studies suggest that families should act as a facilitator and provide needed information for women with ID so that they can make their own choices and develop their autonomy [46, 56, 57]. However, some studies note that families cannot provide sexuality education for women with ID due to sociocultural barriers. Hence sex education approaches need to occur in multiple venues, including schools and healthcare arenas. For example, the findings of Kwai-sang Yau et al. indicate that some parents forbid their children from seeing passionate or erotic scenes in movies by turning the television off [44]. It seems that parents and carers can have an informative discussion with the adult with ID about passionate scenes instead of forbidding them.

In addition, because of cognitive limitations, women with ID are more vulnerable to risks like sexual exploitation, unwanted pregnancy and STI than their nondisabled peers. This issue can be investigated from different dimensions. Self-protection skills such as consent to sex and reporting the sexually abusive incident should be taught by public or non-governmental organizations (NGOs) for women with ID [58]. In the included studies, women with ID had experienced sexual abuse in different contexts. Chamberlain et al. and Elikins et al. report that a 25% and a 27% prevalence of sexual abuse in women with ID in the community and outpatient sections.

Finally, some women with ID talked about their desires for intimate relationships with other women. Specific difficulties such as stigma, lack of training, negative social attitudes, and lack of support by families were experienced by lesbians with ID. Regarding Article 12 of the United Nation Convention on the Rights of People with Disabilities, respecting the decisions of women with ID can be the first key step to address this challenge [59]. Also, cultural provisions and religious constraints are two notable barriers to the sexual expression of homosexual people with ID that should be considered in future research and policy endeavors.

### Limitations

Our study face some limitations. This study only represented the sexual health concerns of women with ID, while females with ID aged under 16 years and other women with disabilities (e.g. hearing impairments, physical disabilities, mental disorders and vision loss) may have different concerns and needs that can be investigated in future studies. We did not include gray literature and non-English language articles which in turn may affect the findings of the present study. Our study did not examine the sexual health concerns in men with ID. Men with ID may have complex and unique needs and concerns due to different psychological, biological, cultural and environmental factors that can be investigated in future studies [8, 60]. To look for more relevant articles, and regarding that the databases had different features to search data, we used different search terms to find the included articles. Furthermore, it should be noted that we included papers published from January 2000 to December 2017. We think that the coronavirus pandemic was one of the main reasons for the delay in the peer review process.

### Implication for practice and/or policy

Participating in educational and supportive programs can help promote constructive options to develop healthy relationships for women with ID. Families need to provide more opportunities for women with ID to express their feelings. Families should act as a facilitator and provide needed information for women with ID so that they can make their own choices and develop their autonomy. Respecting the feelings and desires of women with ID should be considered by families and policymakers. Cultural provisions and religious constraints are notable barriers to the sexual expression of homosexual people with ID that should be considered in future research and policy endeavors.

## Conclusion

In general, participants with ID mentioned various concerns such as lack of sexual experience, negative experiences, lack of understanding, problem with finding a right partner, lack of access to information, sexual abuse, and limited knowledge of sexual behaviors. The findings indicate that women with ID need to be provided with school-based sexuality education tailored to the level of understanding needed to attain the requisite knowledge to form relationships, understand sexual and romantic relationships, and practice safe sex when they choose this option. Families along with education and healthcare systems should provide opportunities for women with ID to talk about their sexual needs and make their own choices.

## Data Availability

Dr. Soltani have full access to all the data in the study and takes full responsibility for the integrity of the data and the accuracy of the data analysis.

## Abbreviations

ID: Intellectual disability
COREQ: Consolidated Criteria for Reporting Qualitative Research
PRISMA: Preferred Reporting Items for Systematic Reviews and Meta-Analysis
WHO: World Health Organization
STI: Sexually transmitted infections
NGO: Non-governmental organizations
